# Incidence of thyroid adverse events following immune checkpoint inhibitor treatment in patients with baseline positive thyroid antibodies: a systematic review and meta-analysis

**DOI:** 10.3389/fonc.2025.1583592

**Published:** 2025-07-23

**Authors:** Hui Yu, Qichao Chen, Wenbing Hu, Yuanhao Chen, Hui Ming

**Affiliations:** ^1^ Department of Nuclear Medicine, Huangshi Central Hospital, Affiliated Hospital of Hubei Polytechnic University, Huangshi, China; ^2^ Department of Oncology, Huangshi Central Hospital, Affiliated Hospital of Hubei Polytechnic University, Huangshi, China

**Keywords:** immune checkpoint inhibitors, thyroid antibodies, thyroglobulin antibody, thyroid peroxidase antibody, thyroid-related adverse events, thyroid dysfunction

## Abstract

**Background:**

To systematically evaluate the incidence of thyroid adverse events in patients with baseline positive thyroid antibodies following treatment with immune checkpoint inhibitors (ICIs).

**Methods:**

In this systematic review and meta-analysis, we utilized PubMed, Embase, and Cochrane Library databases to identify studies that detail the thyroid immune-related adverse events (irAEs) among cancer patients undergoing treatment with ICIs. Literature was screened according to inclusion and exclusion criteria, and data were extracted. Meta-analysis was conducted using RevMan 5.4 and Stata 16.0 software, with adherence to PRISMA 2020 guidelines.

**Results:**

A total of 13 studies involving 2,059 patients treated with ICIs for malignancies were included, with 594 cases of thyroid irAEs reported post-treatment. Meta-analysis revealed that the incidence of thyroid irAEs in patients with baseline positive thyroid antibodies was 66.7% (95% CI: 45.1%, 85.5%; Z=7.825, *p*<0.001). Subgroup analysis indicated that heterogeneity was influenced by geographic region, tumor type, and study type. In an exploratory analysis of 4 studies, thyroglobulin antibody (TgAb) positivity showed a numerically higher risk (but statistically non-significant) of thyroid irAEs compared to thyroid peroxidase antibody (TPOAb) positivity (OR=1.83, 95% CI: 0.87–3.85; Z=1.58, *p*=0.114), but the small number of studies precludes definitive conclusions.

**Conclusion:**

Patients with baseline positive thyroid antibodies experience a higher incidence of thyroid irAEs following ICI treatment. In comparison to TPOAb, baseline TgAb positivity showed a non-significant trend toward higher thyroid irAE risk (based on limited studies), but further evidence is needed to confirm this relationship.

**Systematic review registration:**

https://www.crd.york.ac.uk/PROSPERO/, identifier CRD42025635209.

## 1 Introduction

Immune checkpoint inhibitors (ICIs) targeting cytotoxic T-lymphocyte-associated protein 4 (CTLA-4), programmed cell death protein 1 (PD-1), and programmed death-ligand 1 (PD-L1) have emerged as a pivotal therapeutic modality in the clinical management of malignancies. By inhibiting the mechanism by which cancer cells evade host T-cells, ICIs play a significant role in improving survival rates in patients with advanced solid tumors (1). While activating host T-cells to target antigens, the blockade of inhibitory checkpoints may lead to attacks on other tissues, resulting in immune system activation that also targets the body’s own organs (2).

Consequently, ICI treatment can lead to immune-related adverse events (irAEs), among which thyroid irAEs are particularly prevalent. In most cases, thyroid irAEs manifest as transient thyrotoxicosis, followed by hypothyroidism, resembling the course of classic thyroiditis, though the exact cause remains unclear (3). Studies have found that thyroid autoantibodies are significantly elevated in patients who develop thyroid dysfunction after ICI treatment (4). Baseline thyroid peroxidase antibody (TPOAb) and thyroglobulin antibody (TgAb) positivity may serve as valuable predictive biomarkers for identifying the risk of thyroid irAEs (5). However, two critical gaps remain: (1) the magnitude of this risk has not been systematically quantified in a meta-analytic framework, and (2) whether the risk differs by antibody subtype (TPOAb vs. TgAb) is unclear. Our study addresses these gaps by providing pooled risk estimates and stratifying analyses by antibody type, offering new insights for risk stratification.

Consequently, with the intention of conducting a thorough assessment of the importance of baseline thyroid antibodies and presenting well-grounded medical insights to aid in clinical decision-making, we carried out a painstaking meta-analysis of the published literature.

## 2 Materials and methods

The study protocol was registered in PROSPERO (CRD42025635209) prior to data extraction to minimize bias and ensure methodological rigor.

### 2.1 Search strategy

We conducted a computerized search of PubMed, Embase, and Cochrane Library databases. Search terms included: “Immune Checkpoint Inhibitors,” “PD-1 Inhibitors,” “Programmed Cell Death Protein 1 Inhibitors,” “PD-L1 Inhibitors,” “Programmed Death-Ligand 1 Inhibitors,” “CTLA-4 Inhibitors,” “Cytotoxic T-Lymphocyte-Associated Protein 4 Inhibitors,” “nivolumab,” “pembrolizumab,” “durvalumab,” “avelumab,” “atezolizumab,” “ipilimumab,” “tremelimumab,” “cemiplimab,” “camrelizumab,” “sintilimab,” “tislelizumab,” “toripalimab,” “cancers,” “tumors,” “malignancies,” “malignant neoplasms,” “neoplasm,” “neoplasia,” “thyroid autoantibodies,” “thyroid antibodies,” “thyroglobulin antibodies,” “thyroid peroxidase antibodies.” The database was searched for articles published on or before January 11, 2025. Complete search strategies with all Boolean operators, controlled vocabulary (MeSH/Emtree), and field tags are provided in **Supplementary Table S1**. Two independent reviewers (QC and WH) initially screened all titles and abstracts using Pubmed software. Records were classified as ‘include’, ‘exclude’, or ‘maybe’. Then potentially eligible studies were assessed by the same two reviewers independently. Discrepancies were resolved through discussion, with unresolved cases adjudicated by a third reviewer (YC).

### 2.2 Inclusion and exclusion criteria

The included studies met the following criteria: (1) adult patients diagnosed with advanced, metastatic, or unresectable malignancies, receiving treatment with any ICI, (2) clearly reported thyroid irAEs in the data, (3) measurement of thyroid function and thyroid antibodies (TPOAb and TgAb) before ICI treatment, (4) normal thyroid function in patients before ICI treatment.

Exclusion criteria: (1) studies other than clinical trials such as reviews, case reports, meta-analyses, basic or translational research, and animal studies, (2) literature with unavailable data (including **Supplementary Materials**), (3) studies with fewer than 20 cases of baseline thyroid antibody testing(to balance statistical power with study inclusivity), (4) studies testing only one type of antibody (either TPOAb or TgAb), (5) and duplicate publications.

### 2.3 Data extraction and outcomes

Literature retrieved from the search was imported into Endnote software, and duplicates were removed. Two independent researchers systematically extracted data in accordance with the predefined inclusion and exclusion criteria. A standardized form (**Supplementary Table S2**) was developed. The primary outcome was thyroid irAEs, defined as (1) biochemical thyroid dysfunction (TSH/FT4 abnormalities per study-specific reference ranges), (2) clinically diagnosed thyroid disorders attributed to ICIs, or (c) positive thyroid antibodies (TPOAb/TgAb). Secondary outcomes included time-to-onset of thyroid dysfunction, management strategies (hormone replacement/immunosuppression/ICI modification), and associated survival outcomes (progression-free survival (PFS) or overall survival (OS)). Extracted variables encompassed study characteristics (design, setting), patient demographics (age, cancer type, ICI regimen), and thyroid-specific parameters (baseline function, antibody status). Outcome definitions were harmonized across studies by prioritizing objective laboratory criteria first, then clinical diagnoses, with discrepancies resolved through independent dual review using predefined categorization rules.

### 2.4 Quality assessment and evidence certainty assessment

The quality of the included studies was assessed using the Newcastle-Ottawa Scale (NOS) for case-control and cohort studies. This instrument assesses the potential for bias due to confounding, in selection of participants, in measurement of interventions, due to departures from intended interventions, due to missing data, and in measurement of outcomes. The total score was 9, with a score ≥6 indicating high-quality literature. Two researchers independently assessed the quality, and disagreements were resolved through discussion. A higher score indicates a lower risk of bias. Certainty of evidence was evaluated through NOS for bias risk, funnel plots/Egger’s test for publication bias, and 95% CI width for precision. Standard GRADE domains requiring comparator data (inconsistency, indirectness) were not applicable.

### 2.5 Statistical analysis

We employed both fixed-effects and random-effects models based on the degree of heterogeneity. Following conventional benchmarks where I² values of 25%, 50%, and 75% represent low, moderate, and high heterogeneity, respectively by Higgins et al. (6), we used the DerSimonian-Laird random-effects model when I² ≥ 50% or when the Q-test p-value was < 0.10. And the source of heterogeneity was further analyzed. Publication bias was assessed using Egger’s regression test. *P* ≥ 0.05 or a 95% CI including 0 indicated no publication bias. *P* < 0.05 was considered statistically significant. All analyses were performed using Stata 16.0, with the ‘metan’ package for meta-analyses.

### 2.6 Ethics statement

This systematic review utilized only publicly available aggregated data from published studies and did not involve access to individual patient records. Therefore, ethical approval was waived by our institutional review board in accordance with the Helsinki Declaration guidelines for secondary research.

## 3 Results

### 3.1 Literature search and screening

**Figure 1:**
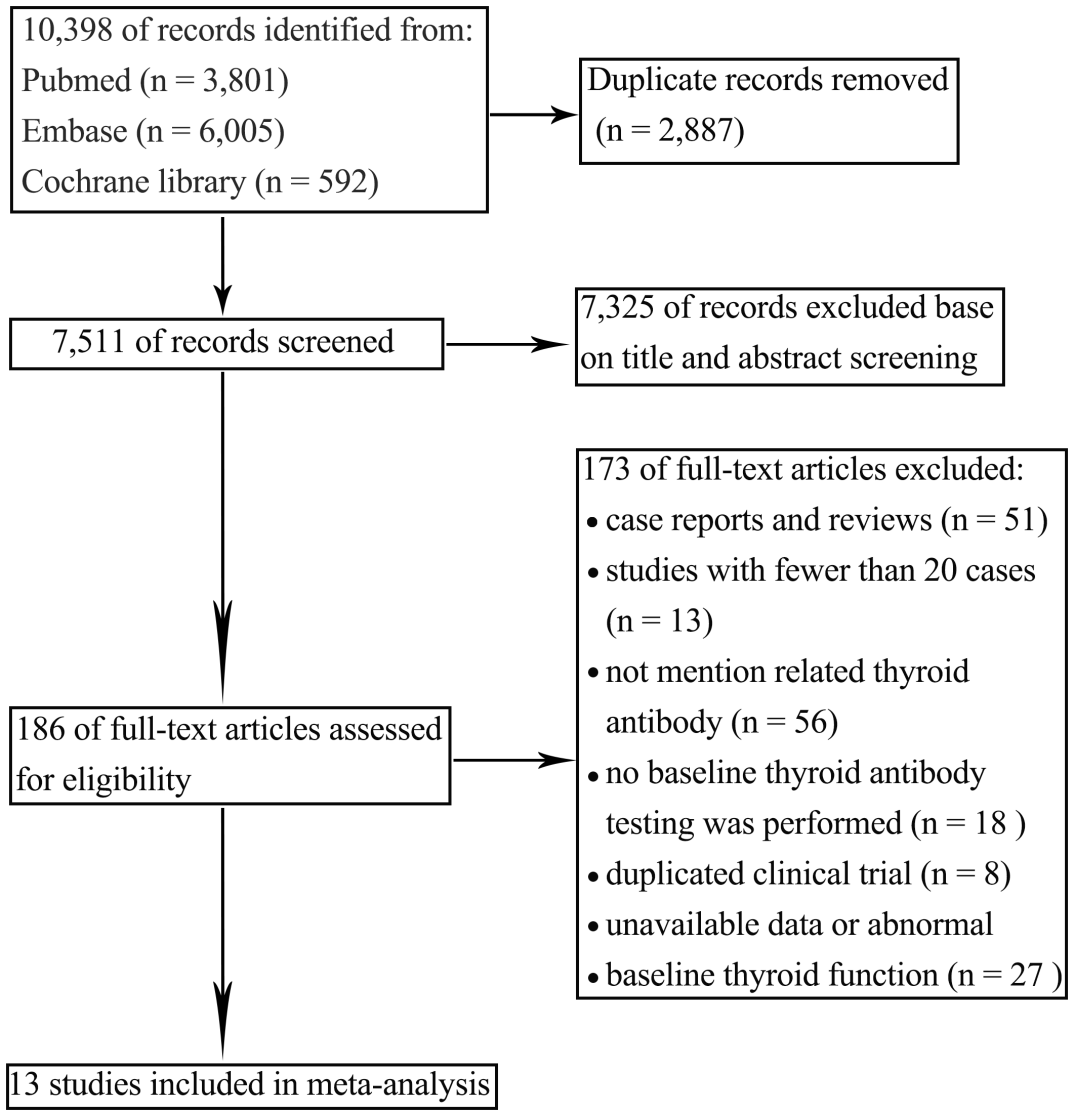
Flow diagram of included studies.

A total of 8,635 articles were initially retrieved, with 6,128 remaining after removing duplicates. Based on a review of titles and abstracts, 168 articles were selected for full-text evaluation. After applying the predefined inclusion and exclusion criteria and excluding studies with abnormal data or incomplete information, 13 articles were ultimately included for qualitative and quantitative analysis. The literature screening process is shown in **Figure 1**.

### 3.2 Basic characteristics of included studies

A total of 13 studies involving 2,059 patients were included, with 594 cases of thyroid irAEs reported post-ICI treatment. Among these, 3 studies reported the incidence of thyroid irAEs in patients who presented with positive baseline TPOAb or TgAb (7–9), One study focused on patients with both TPOAb and TgAb positivity (10), and 9 studies evaluated patients with either TPOAb or TgAb positivity (4, 11–18). The 13 studies were subsequently categorized into two groups: Group A consisted of 10 studies that centered on thyroid antibody positivity (4, 10–18)]. Group B included 4 studies that were dedicated to comparing different types of thyroid antibodies (7–10). The basic characteristics of the included studies are summarized in **Table 1**.

**Table 1:** Characteristics of included studies.

First author	Year	Country	Type of study	Type of tumor	Patients, n	Thyroid irAEs, n*	Baseline positive Thyroid Antibodies, n	Thyroid irAEs, n**	Baseline positive TgAb	Thyroid irAEs, na	Baseline positive TPOAb	Thyroid irAEs, nb
Iwama (4)	2022	Japan	prospective study	Multiple tumors	451	51	100	34	/	/	/	/
Lei (11)	2024	China	retrospective study	Multiple tumors	211	55	58	28	/	/	/	/
Ruggeri (16)	2023	Italy	retrospective study	Multiple tumors	110	32	13	12	/	/	/	/
Takada (15)	2024	Japan	prospective study	Hepatocellular carcinoma	61	2	8	1	/	/	/	/
Gong (12)	2023	China	retrospective study	Multiple tumors	151	56	20	19	/	/	/	/
Luongo (18)	2021	Italy	retrospective study	Multiple tumors	43	9	8	6	/	/	/	/
Muir (17)	2022	Australia	retrospective study	melanoma	122	91	31	30	/	/	/	/
Tang (14)	2022	China	retrospective study	Multiple tumors	146	30	16	12	/	/	/	/
Zhang (13)	2023	China	retrospective study	Multiple tumors	121	58	32	30	/	/	/	/
Kimbara (10)	2018	Japan	retrospective study	Multiple tumors	168	23	35	14	19	12	23	6
Yoon (7)	2021	Korea	retrospective study	Multiple tumors	99	54	/	/	3	2	6	4
Wu (9)	2023	China	retrospective study	Multiple tumors	114	69	/	/	24	20	20	15
Izawa (8)	2022	Japan	retrospective study	Multiple tumors	262	64	/	/	38	15	11	5

^*^Total number of patients with irAEs in the enrolled cohort; ^**^Number of thyroid irAEs in patients with baseline thyroid antibody positivity; ^a^Number of thyroid adverse events in baseline TgAb-positive patients; ^b^Number of thyroid adverse events in baseline TPOAb-positive patients.

irAEs, immune-related thyroid adverse events; TgAb, thyroglobulin antibody; TPOAb, thyroid peroxidase antibody.

### 3.3 Quality assessment results

The NOS quality scores of the included studies are shown in **Table 2**. Overall, the 13 included studies exhibited a high-level quality, with their scores spanning from 6 to 9.

**Table 2:** Quality evaluation.

First author	Type of study	Selection	Comparability	Outcome	Scoring
Iwama (4)	Cohort Study	4	2	1	7
Lei (11)	Case-Control Study	4	2	2	8
Ruggeri (16)	Case-Control Study	4	2	2	8
Takada (15)	Case-Control Study	4	1	2	7
Gong (12)	Case-Control Study	4	2	2	8
Luongo (18)	Case-Control Study	4	2	2	8
Muir (17)	Cohort Study	4	2	2	8
Tang (14)	Cohort Study	4	2	2	8
Zhang (13)	Case-Control Study	4	2	2	8
Kimbara (10)	Case-Control Study	4	2	3	9
Yoon (7)	Case-Control Study	4	1	3	8
Wu (9)	Case-Control Study	4	2	3	9
Izawa (8)	Cohort Study	4	1	2	6

### 3.4 Meta-analysis results

#### 3.4.1 Incidence of thyroid adverse events in group A

**Figure 2:**
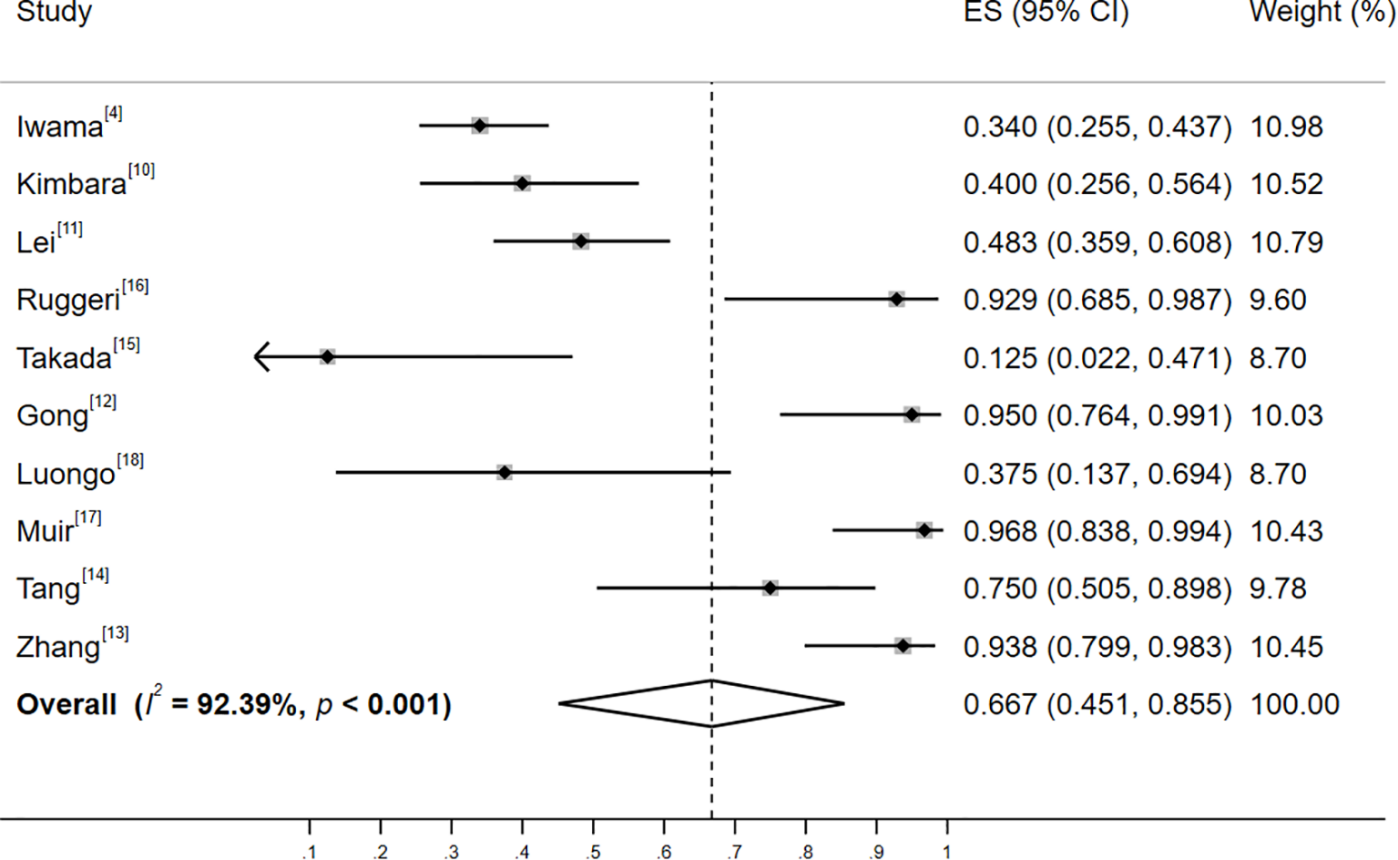
Forest plot of the incidence of thyroid adverse events among patients with positive baseline thyroid antibodies. Heterogeneity: I²=92.39%, *p*<0.001; random-effectss model (DerSimonian-Laird).

Significant heterogeneity was observed among the studies in Group A (I²=92.39%, *p*<0.001), and a random-effects model was employed for the analysis. The results revealed that the incidence of thyroid irAEs in patients with baseline positive thyroid antibodies was 66.7% (95% CI: 45.1%, 85.5%; Z=7.825, *p*<0.001), as illustrated in **Figure 2**. Pre-specified subgroup analyses were performed using random-effects models to assess potential effect modifications by geographic region, tumor type, and study design (see **Supplementary Table S3** for full results). Between-subgroup differences were tested via meta-regression (*p* < 0.05 considered significant). Subgroup analyses revealed the following key patterns:

-Geographic region: In Asian regions, as reported in 7 studies (4, 10–15), the incidence of thyroid adverse events was 55.1%. Conversely, in non - Asian regions, according to 3 studies (16–18), the incidence reached 90.5%.
-Type of tumor: Among studies encompassing multiple tumor types [8 studies (4, 10–14, 16, 18)], the incidence of thyroid adverse events was 61.2%. In contrast, for studies focusing on single tumor type [2 studies (15, 17)] the incidence was 87.5%.
-Type of study: In prospective studies [2 studies (4, 15)], the incidence of thyroid adverse events was 45.3%. In retrospective studies [8 studies (10–14, 16–18)], the incidence was 66.8%.

#### 3.4.2 Risk of thyroid adverse events in group B

**Figure 3:**
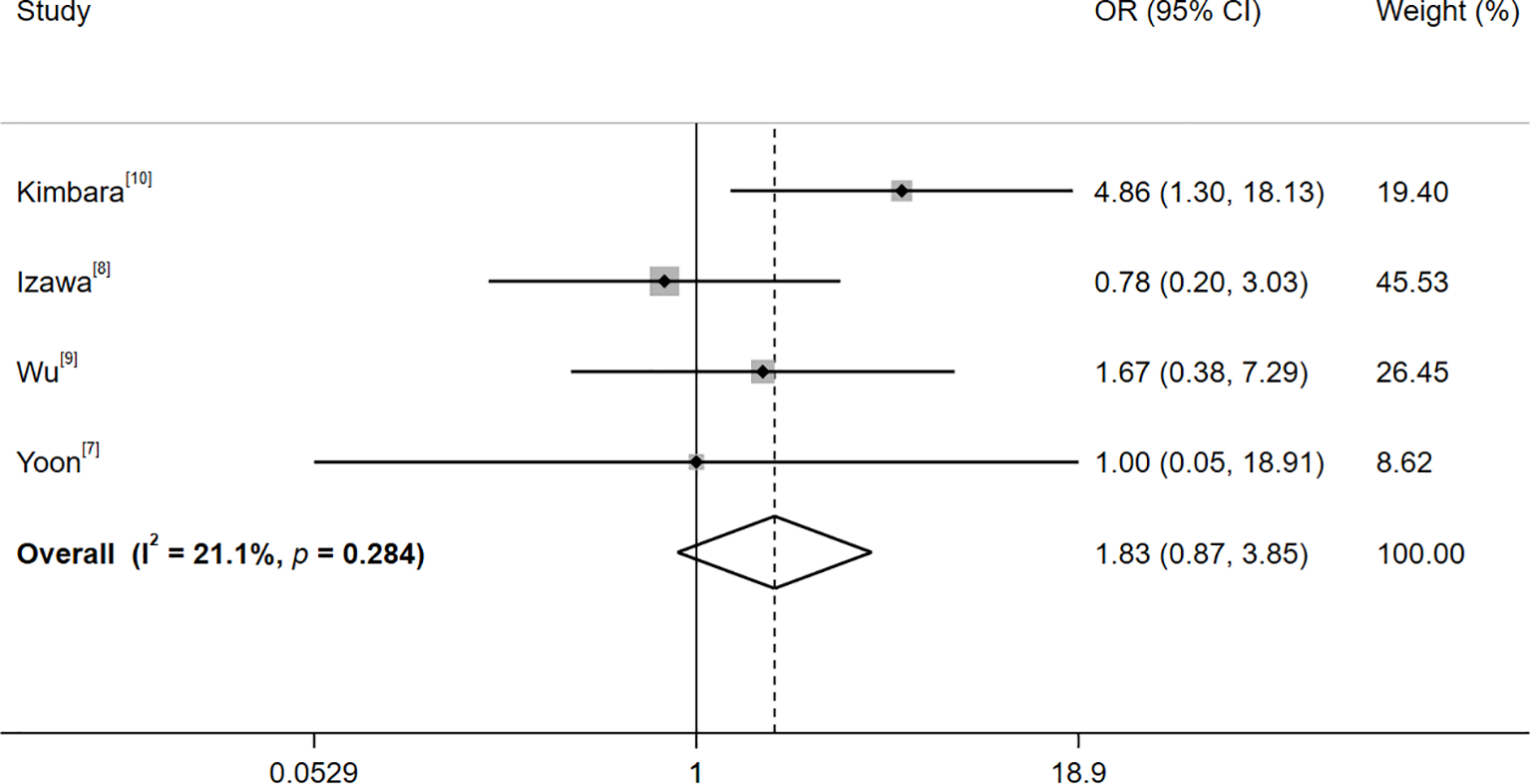
Forest plot of the risk of thyroid adverse events after treatment with immune checkpoint inhibitors with baseline TgAb and TPOAb positivity. Heterogeneity: I²=21.1%, *p*=0.284; fixed-effectss model (Mantel-Haenszel); OR > 1 favors TgAb+ for thyroid irAEs.

No significant heterogeneity was observed among the studies in Group B (I²=21.1%, *p*=0.284). Consequently, a fixed-effects model was employed. The outcomes, as presented in **Figure 3**, demonstrated that, in comparison to the positivity of TPOAb, the baseline positivity of TgAb was associated with a relatively higher risk of thyroid adverse events (OR=1.83, 95% CI: 0.87–3.85; Z=1.58, *p*=0.114). Nevertheless, this difference did not reach statistical significance.

#### 3.4.3 Publication bias

Publication bias was evaluated through Egger’s regression test. In Group A (characterized by thyroid antibody positivity), Egger’s test indicated no significant publication bias (*p* = 0.272; 95% CI: -1.532, 4.742). A funnel plot was generated (**Supplementary Figure S1**) and showed symmetrical distribution, further supporting the absence of publication bias in this group. Sensitivity analysis (**Supplementary Figure S2**) demonstrated stable pooled estimates when any single study was iteratively excluded, confirming that no individual study disproportionately influenced the overall effect.

For Group B (defined by different thyroid antibody types), the Egger’s test yielded a p-value of 0.677 (95% CI: -9.236, 11.574). However, due to the limited number of included studies (n=4), the statistical power of this analysis is substantially reduced. According to the Cochrane Handbook, publication bias assessments are generally unreliable when fewer than 10 studies are included. Thus, while no statistical evidence of bias was detected, this result should be interpreted with caution given the small sample size.

#### 3.4.4 Certainty of evidence synthesis

Evidence certainty summaries are presented in **Table 3**. For incidence of thyroid irAEs, evidence certainty was rated as ‘moderate’ due to imprecision (95% CI width=40.4%, exceeding our pre-defined threshold of 15%), despite low bias risk (all studies scored NOS≥7) and no significant publication bias (Egger’s test p ≥ 0.05).

**Table 3:** Evidence certainty assessment for primary outcomes.

Outcome	Pooled Rate (95%CI)	Bias Risk	Imprecision	Pub. Bias	Certainty
Thyroid irAEs	66.7% (45.1-85.5%)	Low	Yes	No	Moderate

Certainty levels: High (no limitations), Moderate (one downgrade), Low (two downgrades), Very Low (three downgrades).

## 4 Discussion

Thyroid dysfunction is a relatively common adverse event following ICI treatment for malignancies, with hypothyroidism being the most frequently reported manifestation. The role of thyroid autoantibodies in the pathogenesis of ICI-related thyroid irAEs remains incompletely understood (19). However, studies have demonstrated an association between baseline TPOAb and TgAb positivity and the development of thyroid irAEs (20). Higher initial positivity rates and post-treatment seroconversion rates of thyroid antibodies in the thyroid irAE group compared to the non-thyroid irAE group (23.8% vs. 4.9% and 42.99% vs. 9.8%, respectively), along with higher initial TPOAb titers (3.490 vs. 0.935 IU/mL), although TgAb titers did not differ significantly (11). However, clinically meaningful thresholds for antibody titers remain undefined, limiting their predictive utility. Following ICI treatment, seroconversion or an increase in the titers of TPOAb and/or TgAb has been found to be associated with thyroid irAEs (12). Notably, thyroid irAEs can also occur in patients with negative thyroid antibodies (16).

This meta-analysis included 13 studies involving 2,059 patients (with 594 cases of thyroid irAEs reported post-ICI treatment), demonstrated that 69.1% of patients with pre-existing thyroid autoantibodies developed thyroid irAEs. The high incidence rate suggests that routine thyroid antibody testing before initiating ICI therapy could help identify high-risk patients. While universal screening may not be cost-effective in all settings, targeted testing should be considered for patients with a personal or family history of autoimmune thyroid disorders, populations with a higher prevalence of thyroid autoimmunity, and those receiving combination ICI regimens (e.g., anti-CTLA-4 plus anti-PD-1), which carry higher thyroid irAE risks. For antibody-positive patients, we recommend that baseline and serial monitoring of thyroid function tests every 4-6 weeks during treatment and proactive patient education about symptoms of thyroid dysfunction. These findings support the potential clinical utility of pre-ICI thyroid antibody screening, particularly in high-risk subgroups. Future studies should evaluate the cost-effectiveness of such targeted testing and its impact on early intervention outcomes.

Subgroup analyses revealed higher incidences in non-Asian regions, studies involving multiple tumor types, and retrospective studies compared to Asian regions, single tumor types, and prospective studies, potentially explaining these discrepancies.

Studies indicate that both TPOAb and TgAb are implicated in the thyroid destruction observed in Hashimoto’s thyroiditis, and TgAb may exert a more substantial influence than TPOAb in PD-1-induced destructive thyroiditis (10–12). Muir et al. revealed that in patients manifesting overt thyrotoxicosis, the presence of positive TgAb could potentially serve as an indicator of an elevated probability of progressing to permanent hypothyroidism (17). Notably, the co-presence of positive TPOAb and TgAb is especially pronounced among patients experiencing overt thyroid dysfunction. In our study, the comparison between TgAb and TPOAb positivity was based on only 4 studies, and the lack of statistical significance (p=0.114) may reflect limited statistical power rather than a true absence of association. Future larger-scale studies are needed to validate these preliminary findings.

The baseline levels of thyroid-stimulating hormone (TSH) could potentially be correlated with the onset of hypothyroidism subsequent to the administration of ICI. This observation implies that ICI treatment is more likely to expedite the advancement of subclinical Hashimoto’s thyroiditis, rather than triggering *de novo* ICI-mediated thyroiditis (20). In the multivariate analysis conducted by Kimbara, it was revealed that TSH levels (≥5 mIU/L) and the presence of positive TgAb exhibited a significant association with irAEs(OR 7.36%, 95% CI: 1.66–32.7, *p*=0.01; OR 26.5%, 95% CI: 8.18–85.8; *p*<0.001) (10). Meanwhile, Luongo et al. reported that among 20 patients with a baseline TSH level of at least 1.67 mIU/L, 13 of them developed hypothyroidism during the initial four-month of ICI treatment (18).

In a retrospective study carried out by Pollack (21), it was discovered that baseline TSH levels were significantly higher in patients who developed hypothyroidism compared to those with hyperthyroidism or normal thyroid function (2.85 ± 1.85 vs. 1.75 ± 1.29 vs. 1.99 ± 1.95 mU/L, *p*=0.021), with a baseline TSH >2.19 mU/L associated with an increased risk of overt thyroid dysfunction. Thyroid irAEs could be correlated with the prognosis of patients. Intriguingly, research has demonstrated that among non-small cell lung cancer patients undergoing treatment with ICIs, individuals experiencing thyroid dysfunction exhibited a notable improvement in both PFS (HR=0.54, 95% CI: 0.44–0.64) and OS (HR=0.34, 95% CI: 0.25–0.44), with a 66% reduction in the risk of death and a 46% reduction in the risk of disease progression (22). Cheung et al. (23) similarly reported longer PFS and OS in lung cancer and melanoma patients who developed thyroid irAEs. The consistent association between thyroid irAEs and survival likely stems from shared upstream mechanisms. ICI-induced T-cell activation drives both anti-tumor immunity and autoimmune toxicity (including thyroiditis) (24). Thyroid irAEs may thus serve as a “bystander biomarker” of effective systemic immune response, rather than directly causing survival benefits (25). Consequently, the presence of positive thyroid antibodies at baseline might suggest more favorable prognoses for cancer patients undergoing ICI therapy. Moreover, this baseline thyroid antibody positivity has the potential to serve as a valuable biomarker for forecasting the clinical response subsequent to ICI treatment.

This study is subject to several limitations. To begin with, the small sample size (n=4 studies) comparing thyroid antibody subtypes may increases susceptibility to bias. The exclusion of studies with <20 baseline thyroid antibody tests may introduce selection bias, as smaller studies might report different antibody-irAE associations. Secondly, the analysis was significantly constrained by the unavailability of individual patient data, which prevented (1): quantitative assessment of thyroid antibody titers and establishment of clinically relevant cutoff values, and (2) detailed temporal analyses including dose-response relationships and precise time-to-onset determinations for thyroid irAEs. This limitation fundamentally restricted our ability to characterize potential threshold effects or progression patterns of thyroid autoimmunity following ICI exposure. Therefore, prospective studies are needed to establish validated cutoff values (e.g., fold-change from baseline or absolute thresholds), and to determine whether antibody changes precede or follow thyroid dysfunction. Moreover, it becomes infeasible to conduct a comparison of the incidence of thyroid irAEs between patients with single antibody positivity and those with dual antibody positivity. Finally, methodological heterogeneity may affect result generalizability, including: (1) combined analysis of randomized and observational studies introducing design heterogeneity; (2) variable irAE definitions potentially affecting cross-study comparability; (3) limited subgroup analyses (n ≤ 3 studies) where heterogeneity assessment was unreliable; (4) potential residual confounding from differences in ICI regimens, cancer types, and dosing schedules; (5) possible exclusion of relevant non-English literature. In light of the current findings and limitations, it is imperative that further in-depth studies be carried out.

In summary, patients who present with positive thyroid antibodies at the baseline stage exhibit a relatively higher incidence of thyroid irAEs subsequent to ICI treatment. While baseline TgAb positivity appears to confer a slightly higher risk compared to TPOAb positivity, the difference is not statistically significant. Further studies are warranted to refine risk stratification and optimize the management of thyroid irAEs in patients receiving ICI therapy.

## Data Availability

The raw data supporting the conclusions of this article will be made available by the authors, without undue reservation.
