# Supplementary material for: Incidence of thyroid adverse events following immune checkpoint inhibitor treatment in patients with baseline positive thyroid antibodies: a systematic review and meta-analysis
Source: Front Oncol. 2025 Jul 23;15:1583592. doi: 10.3389/fonc.2025.1583592 (PMC12325276; doi:10.3389/fonc.2025.1583592)
Supplement: Supplementary file 1 [file DataSheet1.doc]

Supplement Figures and Tables

Figure S1. Funnel plot


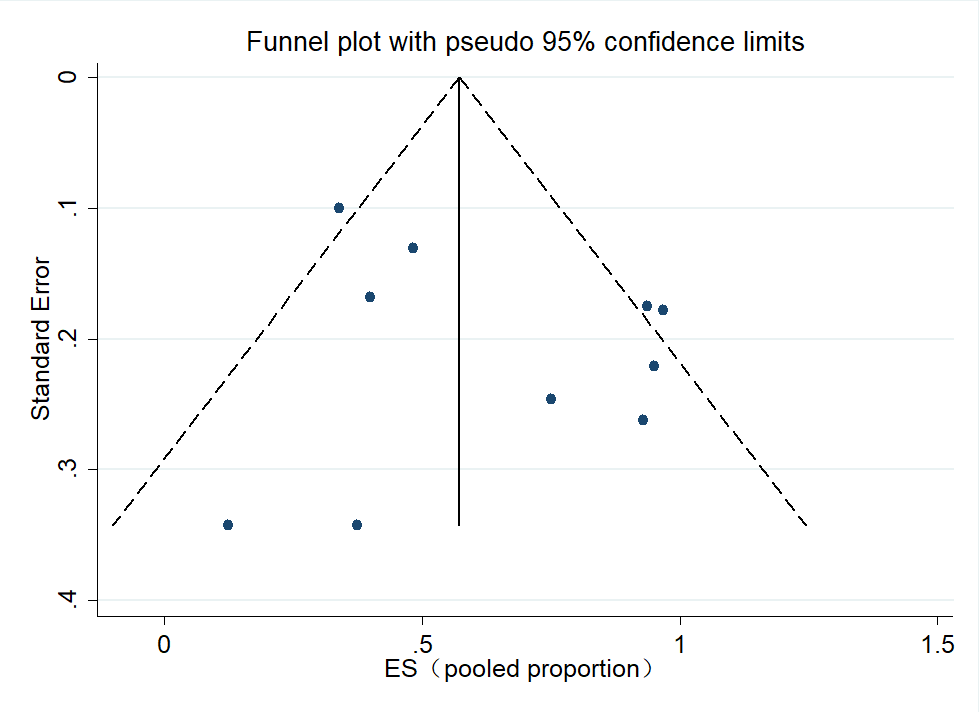


Figure S2. Sensitivity analysis


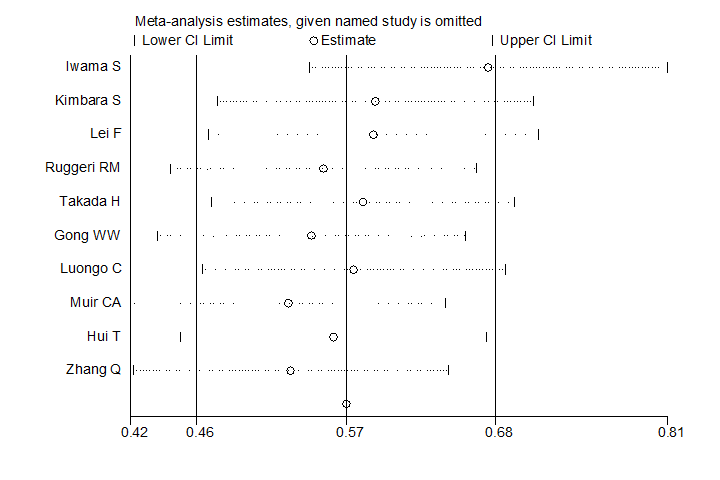


Table S1: Search methods

| Steps | Search methods |
| --- | --- |
| #1 | "Immune Checkpoint Inhibitors"[MeSH Terms] OR "Immune Checkpoint Blockers"[All fields] OR "Immune Checkpoint Blockade"[All fields] OR "Immune Checkpoint Inhibition"[All fields] OR "PD-L1 Inhibitors"[All fields] OR "Programmed Death-Ligand 1 Inhibitors"[All fields] OR "PD-1-PD-L1 Blockade"[All fields] OR "CTLA-4 Inhibitors"[All fields] OR "Cytotoxic T-Lymphocyte-Associated Protein 4 Inhibitors"[All fields] OR "PD-1 Inhibitors"[All fields] OR "Programmed Cell Death Protein 1 Inhibitor"[All fields] OR "Nivolumab"[All fields] OR "Pembrolizumab"[All fields] OR "Durvalumab"[All fields] OR "Avelumab"[All fields] OR "Atezolizumab"[All fields] OR "Ipilimumab"[All fields] OR (Tremelimumab[All fields] OR "Cemiplimab"[All fields] OR "Camrelizumab"[All fields] OR "Sintilimab"[All fields] OR "Tislelizumab"[All fields] OR "Toripalimab"[All fields] |
| #2 | "Thyroid Autoantibodies"[All fields] OR "Thyroid Autoantibody"[All fields] OR "thyroid dysfunction"[All fields] OR "hyperthyroidism"[All fields] OR "hypothyroidism"[All fields])) OR "thyroiditis"[All fields] OR "thyroid immune-related adverse events"[All fields] OR "immune-related adverse events"[All fields] |
| #3 | "Neoplasms"[MeSH Terms] OR "Tumor"[All fields] OR "Neoplasia"[All fields] OR "Cancer"[All fields] "Malignant Neoplasm"[All fields] OR "Malignancy"[All fields] OR "Benign Neoplasms"[All fields] |
| #4 | #1 AND #2 AND #3 |
| #5 | #4 Filters: human |

Table S2: Standardized Data Extraction Form for Systematic Review

| Field Category | Specific Items | Definition/Harmonization Strategy |
| --- | --- | --- |
| 1. Study Identification | First author, publication year | _ |
|  | Country/Region | _ |
| 2. Study Characteristics | Design (RCT/Cohort/Case-control/prospective/retrospective) | _ |
|  | Sample size (Total/Intervention/Control) | _ |
| 3. Patient Demographics | Mean age (SD) or median (IQR) | Converted to years; if ranges given, calculate midpoint |
|  | Sex distribution (% male/female) | _ |
|  | Cancer type and stage | Coded by ICD-O-3; stage per AJCC 8th edition 2 |
|  | Baseline comorbidities (e.g., autoimmune disease) | Listed and grouped by system |
| 4. Intervention Details | ICI drug class (PD-1/PD-L1/CTLA-4) | Single/Combination therapy specified |
|  | Treatment duration (weeks) | _ |
|  | Concomitant therapies (Chemo/Targeted) | Yes/No; drug names recorded |
| 5. Thyroid Parameters | Baseline thyroid function (TSH/FT4/FT3) | _ |
|  | Baseline thyroid antibody (TPOAb/TgAb ) | Antibody assay method (ECLIA/RIA/Other); Antibody positivity threshold |
|  | Thyroid irAE definition | Prioritized hierarchy: CTCAE v5.0 criteria; Study-specific lab criteria (TSH/FT4/FT3); Clinician diagnosis |
| 6. Outcome Data | Thyroid irAE incidence rate (%) | Calculated as events/total patients |
|  | Time-to-onset (days post-ICI) | Median (IQR) or mean (SD); if Kaplan-Meier curve, extract survival data |
|  | Management strategy (Levothyroxine/Glucocorticoids/ICI discontinuation) | Categorization: Hormone replacement: Levothyroxine; Immunosuppression: Prednisone >10mg/day >1 week; ICI modification: Delay/Stop |
|  | Survival outcomes (PFS/OS) | Adjusted HR (95% CI) extracted; covariates recorded (e.g., age, stage) |
| 7. Risk of Bias (NOS) | Key concerns (e.g., selection bias) | Summarized per domain |

Table S3: Subgroup Analysis of Incidence of Thyroid Adverse Events

| Subgroup | Studies (n) | Effect Size (95% CI) | I² (%) | Z | p value |
| --- | --- | --- | --- | --- | --- |
| Region |  |  |  |  |  |
| Asian | 7 | 0.551 (0.316–0.776) | 91.72% | 6.196 | 0.001 |
| non - Asian | 3 | 0.905 (0.749–0.996) | —* | 11.218 | 0.001 |
| Type of Tumor |  |  |  |  |  |
| multiple tumor types | 8 | 0.612 (0.368–0.831) | 93.03% | 6.521 | 0.001 |
| single tumor type | 2 | 0.875 (0.739–0.971) | —* | 12.966 | 0.001 |
| Type of Study |  |  |  |  |  |
| prospective study | 2 | 0.453 (0.363–0.544) | —* | 14.379 | 0.001 |
| retrospective study | 8 | 0.875 (0.739–0.971) | 90.21% | 7.082 | 0.001 |
| Overall Effect | 10 | 0.667(0.451–0.855) | 92.39% | 7.825 | 0.001 |
| *Heterogeneity statistics (I²) were not calculated for subgroups with fewer than 4 studies, as these estimates become unreliable with small sample sizes | | | | | |
